# Myosin X is required for efficient melanoblast migration and melanoma initiation and metastasis

**DOI:** 10.1038/s41598-018-28717-y

**Published:** 2018-07-11

**Authors:** Hiroshi Tokuo, Jag Bhawan, Lynne M. Coluccio

**Affiliations:** 1 Department of Physiology & Biophysics, Boston University School of Medicine, Boston, MA 02118 USA; 2 Department of Dermatology, Boston University School of Medicine, Boston, MA 02118 USA

## Abstract

Myosin X (Myo10), an actin-associated molecular motor, has a clear role in filopodia induction and cell migration *in vitro*, but its role *in vivo* in mammals is not well understood. Here, we investigate the role of Myo10 in melanocyte lineage and melanoma induction. We found that Myo10 knockout (Myo10KO) mice exhibit a white spot on their belly caused by reduced melanoblast migration. Myo10KO mice crossed with available mice that conditionally express in melanocytes the BRAF^V600E^ mutation combined with Pten silencing exhibited reduced melanoma development and metastasis, which extended medial survival time. Knockdown of Myo10 (Myo10kd) in B16F1 mouse melanoma cell lines decreased lung colonization after tail-vein injection. Myo10kd also inhibited long protrusion (LP) formation by reducing the transportation of its cargo molecule vasodilator-stimulated phosphoprotein (VASP) to the leading edge of migrating cells. These findings provide the first genetic evidence for the involvement of Myo10 not only in melanoblast migration, but also in melanoma development and metastasis.

## Introduction

Myo10 is one of several actin-based motor molecules in the myosin superfamily. It has a motor or head domain with a nucleotide-binding site and an actin-binding site, an IQ or neck domain, which binds three molecules of calmodulin, and a C-terminal tail domain that has a single α-helix (SAH) region followed by a coiled-coil region presumably involved in dimerization, 3 PEST sequences, which are rich in proline, glutamate, serine and threonine and confer sensitivity to certain proteases, 3 pleckstrin homology (PH) domains, a Myosin Tail Homology 4 (MyTH4) domain, which binds to microtubules, and a band 4.1, Ezrin, Radixin, Merlin (FERM) domain^1^.

Myo10 localizes to the tips of filopodia, actin-rich finger-like protrusions found at the leading edge of cells^2^ and believed to be involved in numerous cellular processes including cell migration, wound healing, adhesion to the extracellular matrix, guidance towards chemoattractants, neuronal growth-cone path finding and embryonic development^3^. Presumably, Myo10 exists chiefly as a folded monomer in the cytoplasm; however, contact with phosphatidylinositol (3,4,5)-triphosphate (PIP_3_) induces its unfolding and dimerization to act as a processive motor able to transport cargo^4^. Previous studies have shown that Myo10 promotes filopodia formation by delivering specific cargos to the cell periphery, e.g., Mena/VASP, which binds to the tail domain^5,6^, and integrins^7^, netrin receptors^8^, and VE-cadherin^9^, which bind to the FERM domain. Also, the PH domain of Myo10 binds to PIP_3_ and targets Myo10 to the cell membrane of the leading edge for filopodia formation^10^.

Myo10 is expressed ubiquitously in vertebrate tissues^2,11,12^, and its functional importance has been found in a variety of cells/tissues, e.g., phagocytosis cup formation in leukocytes^13^, nuclear anchoring and spindle assembly in *Xenopus laevis*^14^, orientation of the mitotic spindle in cultured cells^15^, endothelial cell migration and angiogenesis^16^, axonal path-finding regulated by netrin^8^, cranial neural crest cell migration in *Xenopus*^17,18^, sealing zone patterning^19^ and differentiation^20^ in osteoclasts, tight junction barrier formation in polarized epithelial cells^21^, axon outgrowth in cortical neurons^22^, formation of *Shigella*-induced membrane protrusions and cell-to-cell spreading^23^, dendritic spine development by VASP trafficking^6^, and leukocyte extravasation through cultured endothelial cells^24^. Most recently, reduced filopodia formation was observed in isolated macrophages from Myo10KO mice^25^ and in retinal angiogenesis in the eyes of Myo10KO mice^26^. In spite of these studies, the physiological and pathological functions of Myo10 in murine or human organs is still largely unknown.

Recently, information regarding the role of Myo10 in cancer has been accumulating, e.g., the expression level of Myo10 correlates with aggressiveness and metastasis of breast cancer^27,28^; Myo10 is up-regulated and participates in lung adenocarcinoma metastasis^29^; Myo10 was found as a target of microRNA-124, which was suppressed in aggressive non-small cell lung cancer^30^; Myo10 was also a target of microRNA-340, which inhibits the metastasis of breast cancer^31^; MYO10 expression was higher in prostate cancer than in normal tissue; and Myo10kd reduced the migration speed and directional persistence of a prostate cancer cell line^32^.

In this study, we found that Myo10KO mice (*Myo10*^*tm2/tm2*^) exhibit a white belly spot and syndactyly, striking defects that were also reported with other types of Myo10KO mice (*Myo10*^*tm1d/tm1d*^, *Myo10*^*tm1a/tm1a*^, and *Myo10*^*m1J/m1J*^) while this paper was in review^26^. Our focus is on the white belly spot (which is observed in all of the Myo10KO mice) for the following reasons: (i) Natural mouse mutants with white belly spots have been instrumental to understanding melanoblast (precursors to melanocytes) migration. Studies of *lethal spotting* (*Edn-3*), *piebald* (*Ednrb*), *dominant white spotting* (*Kit*), and *microphthalmia* (*Mitf*) mutants led to the identification of genes and proteins essential for melanoblast migration. For example, the *endothelin-3* (*Edn-3*) ligand, produced by the ectoderm, binds to the *endothelin receptor-B* (*Ednrb*), which is expressed in melanoblasts. Signaling through this complex is essential for migrating melanoblasts to reach their final destination^33^. The kit ligand produced in the dermomyotome, dermis, and hair follicles binds to the c-kit receptor tyrosine kinase, which is expressed by melanoblasts. Only melanoblasts expressing the c-kit and Ednrb receptors respond to the attractive and repulsive signals controlling cell migration^34^. The *Mitf* (microphthalmia-associated transcription factor) gene and the *M-Mitf* isoform (specific to the melanocyte lineage) play a key role in the biology of melanoblasts and melanocytes^35^. The function of this transcription factor is not only to ensure specification and survival of the melanocyte lineage, but also to contribute to their migration through regulation of numerous target genes. Among them a transcription factor, Slug (*Snai2*), is directly regulated by M-Mitf and implicated in melanoblast migration^36^. The transcription factors *Pax3* and *Sox10* synergistically regulate *M-Mitf* expression, and heterozygous mutations in these genes give rise to a white-spotted phenotype^37,38^. Thus, Myo10 may have an important role in melanoblast migration resulting in a white belly spot phenotype. (ii) Recent findings suggest that migration of melanoblasts during development is highly correlative with melanoma metastasis. The imprint of past migratory behavior of melanoblasts has been suggested to confer a propensity of primary melanomas to establish remote metastases^39–41^. Nonetheless, nothing is known about the function of Myo10 in melanoma.

Here, we demonstrate that Myo10KO mice exhibit a white belly spot and show that in cultured melanoblasts Myo10kd decreases the formation of LPs and cell migration. These data indicate that the white belly spot in Myo10KO mice may be a consequence of reduced migration of melanoblasts in the absence of Myo10. We also show that Myo10KO delayed onset and development of melanoma, and reduced metastasis in a mouse melanoma model. We present evidence that Myo10kd in a melanoma cell line greatly impaired pseudopod formation and lung colonization in mice following tail injection. We also determined that elevated expression of the *MYO10* gene was associated with inferior survival outcomes in melanoma patients and Myo10 expression increased in human melanoma. Together, we uncovered for the first time that Myo10-induced protrusions drive melanoblast migration and melanoma initiation and metastasis.

## Results

### Genomic structure of *Myo10*^*tm2(KOMP)Wtsi*^ mice and Myo10 expression

**Figure 1:**
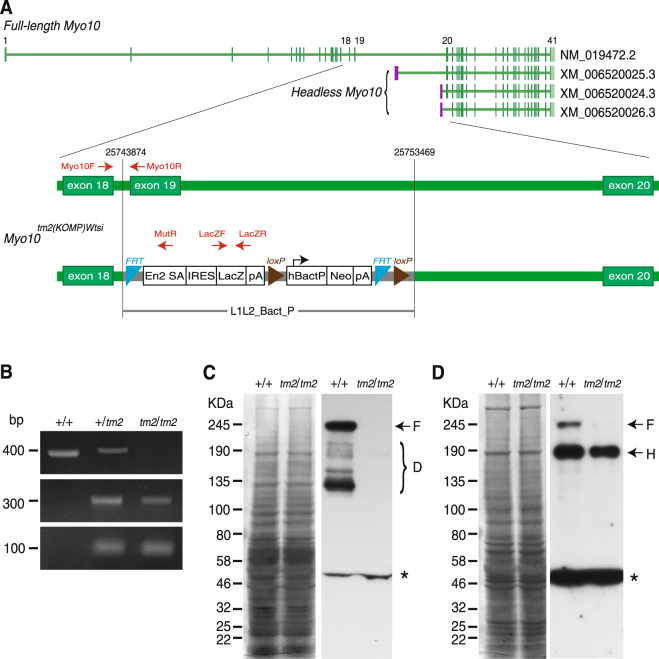
Genomic structure of *Myo10*^*tm2(KOMP)Wtsi*^ mice and expression of Myo10. (**A**) Allele structure of mouse *full-length Myo10* (NM_019472.2) and three alternative splicing variants called “*headless Myo10*” (XM_006520025.3, XM_006520024.3 and XM_006520026.3) (top). Vertical bars on the gene map represent exons, and purple bars represent first exons specific for *headless Myo10*. The lesion from exon 18 to 20 is magnified and primers for genotyping are indicated as red arrows. Genomic structure of *Myo10*^*tm2(KOMP)Wtsi*^ (Myo10KO) mice (bottom). The *Myo10*^*tm2(KOMP)Wtsi*^ allele is shown to illustrate the mutated *Myo10* gene in which the *lacZ* and *neomycin (neo)* expression cassette (L1L2_Bact_P) was inserted to create a deletion 9595 nucleotides in size starting at position 25743874 and ending at position 25753469 of chromosome 15 (Genome Build GRCm38). This deletion results in the removal of functionally critical exon 19 and a portion of intron 19. This cassette contained the splice acceptor of mouse Engrailed 2 exon 2 (En2 SA), an internal ribosome entry sequence (IRES) to initiate *lacZ* translation, and SV40 polyadenylation (pA) to terminate transcription after the *lacZ* gene. The *neo* gene is driven by the human beta actin promoter (hBactP) and contains its own pA. Additionally, L1L2_Bact_P is flanked by FRT (flippase recognition target) sites, and hBactP-neo-pA is flanked by loxP sites. Red arrows indicate the positions of primers for genotyping. The specific details and the complete DNA sequence of the *Myo10*^*tm2(KOMP)Wtsi*^ allele are available on the International Mouse Phenotyping Consortium (IMPC) website (http://www.mousephenotype.org/data/search/allele2?kw=Myo10). (**B**) Characterization of Myo10KO mice by genomic PCR. Genomic DNA samples isolated from WT mice (+/+), mice heterozygous for the Myo10 mutant allele (+/*tm2*), and homozygous mice (*tm2/tm2*) were used as a template for PCR with primer pairs Myo10F/Myo10R, Myo10F/MutR, and LacZF/LacZR. PCR with the primer pair Myo10F/Myo10R produced a 400-bp band (top panel) only in mice that carried at least one copy of the WT allele; PCR with the primer pair Myo10F/MutR resulted in a 295-bp product (middle panel),; and PCR with the primer pair LacZF/LacZR resulted in a 108-bp band (bottom panel) in mice that carried at least one copy of the mutant allele. An image of the whole gel is shown in Supplementary Fig. S1. (**C**,**D**) Immunoblots of tissue samples from WT (+/+) and Myo10 KO (*tm2/tm2*) mice. Kidney (**C**) and brain (**D**) samples were probed using anti-Myo10 antibody (right panels). The probed membranes were stained with CBB (left panels). The asterisk indicates a non-specific band.

To assess Myo10 function *in vivo*, *Myo10*^*tm2(KOMP)Wtsi*^ (Myo10KO) mice were obtained from the Wellcome Trust Sanger Research Institute, and the mouse line was maintained in a C57BL/6 N background. KO was performed by insertion of the L1L2_Bact_P cassette, which created a deletion of 9595 nucleotides starting at position 25743874 and ending at position 25753469 of chromosome 15 (Genome Build GRCm38) (Fig. 1A). This deletion resulted in the removal of exon 19, which is functionally critical, and a part of intron 19, but the alternative transcription start sites and first exons specific for *headless Myo10* remained intact. The precise structure of the *Myo10*^*tm2(KOMP)Wtsi*^ allele is shown in Fig. 1A. The presence and zygosity of the *Myo10*^*tm2(KOMP)Wtsi*^ allele were verified by PCR using genomic DNA isolated from tail snips (Fig. 1B). An image of the whole gel is shown in Supplementary Fig. S1. To confirm the KO effect on protein expression, first we performed immunoblot analysis of whole kidney lysates from one-month old mice and found that only the control mice expressed full-length Myo10 (Fig. 1C, arrow with F). Several extra bands were detected below the molecular weight of full-length Myo10 in wild-type samples only, which we speculate to be degradation products of full-length Myo10 (Fig. 1C, bracket with D). A band (~50 KDa) was found in both genotypes, which is likely a non-specific signal (Fig. 1C, asterisk). Next, to check for expression of headless Myo10, we performed immunoblot analysis of whole brain lysates from one-month old mice, which reportedly express both full-length and headless Myo10^42^. As observed in kidney, full-length Myo10 was detected in brain samples of control mice; however, headless Myo10 was found in brain extracts from both control (+/+) and Myo10KO (*tm2/tm2*) mice (Fig. 1D, arrow with H). A nonspecific band was detected in both genotypes at ~50 KDa as observed in kidney lysates (Fig. 1D, asterisk). These results indicate that the *Myo10*^*tm2(KOMP)Wtsi*^ allele nullifies full-length Myo10, but not headless Myo10.

### Myo10-deficient mice have a white belly spot and syndactyly

**Figure 2:**
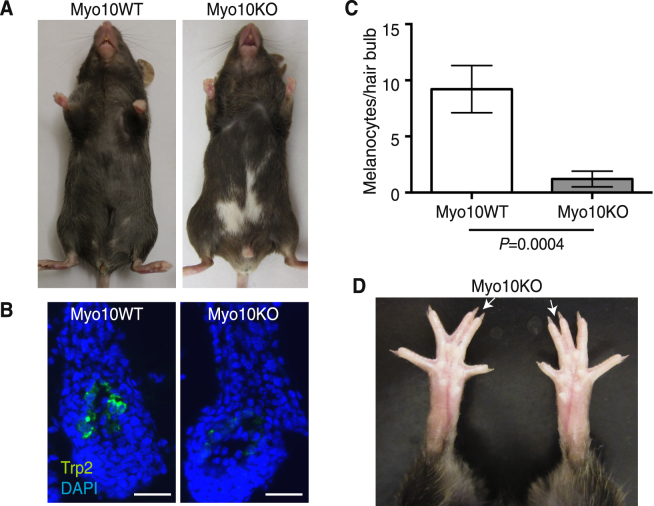
Myo10-deficient mice have a white belly spot and syndactyly. (**A**) Representative coat color of a Myo10KO mouse (right) with control littermate (left, Myo10WT). (**B**) Staining of hair follicle bulbs in the abdominal skin from wild-type and Myo10KO mice for Trp2, a melanocyte marker. Scale bar is 50 μm. (**C**) Quantification of Trp2-positive melanocytes. The average number of Trp2-positive melanocytes in hair bulbs in the white belly spots of Myo10KO mice was 1.2 ± 0.7 (mean ± SEM, 30 hair bulbs per mouse × 3 mice, *n* = 90), which was significantly lower (*P* = 0.0004) than 9.2 ± 2.1 (*n* = 90) for Myo10WT mice. (**D**) Representative image of fused toes (arrows), syndactyly, in Myo10KO mouse.

When heterozygous *Myo10*^+*/tm2*^ mice were crossed with one another, we obtained the following genotypes: 30 *Myo10*^+*/*+^ pups, 80 *Myo10*^+*/tm2*^ pups, and 16 *Myo10*^*tm2/tm2*^ pups. Thus, *Myo10*^*tm2/tm2*^ pups were obtained ~50% as often as wild-type homozygote births, almost the same as the *Myo10*^*tm1d/tm1d*^ and *Myo10*^*tm1a/tm1a*^ pups, which were obtained 40% and 45%, respectively, as often as wild-type homozygote births^26^ (Table 1). We observed a white belly phenotype with 100% penetrance in *Myo10*^*tm2/tm2*^ mice (Fig. 2A), the same rate as in *Myo10*^*tm1a/tm1a*^, *Myo10*^*tm1d/tm1d*^, and *Myo10*^*m1J/m1J*^ mice. Less than 5% of the *Myo10*^*tm2/tm2*^ mice also had white spots along the dorsal midline. Histological analysis of black areas of the *Myo10*^*tm2/tm2*^ mice at P14 showed melanocytes in hair follicles (not shown), whereas the number of melanocytes present in white patches of the *Myo10*^*tm2/tm2*^ mice was reduced by 87% vs. dark patches on control mice (Fig. 2B,C). These results suggest that Myo10 deficiency in the melanocyte lineage causes a pigmentation defect due to the lack of melanocytes in the ventral areas distal to their origin of migration. We also found that *Myo10*^*tm2/tm2*^ mice have webbed digits, a form of syndactyly, in the forelimbs or hind legs with a 72% penetrance rate (Fig. 2D). This rate is slightly higher than that observed in *Myo10*^*tm1a/tm1a*^ (50%), *Myo10*^*tm1d/tm1d*^ (50%), and *Myo10*^*m1J/m1J*^ (43%) mice. We found that only two in 126 (1.6%) *Myo10*^*tm2/tm2*^ mice had a kinked tail, similar to *Myo10*^*m1J/m1J*^ mice, which did not have kinked tails, but a much lower rate than the 75% of *Myo10*^*tm1a/tm1a*^ and the 50% of *Myo10*^*tm1d/tm1d*^ mice that exhibited a visible kink near the tip of the tail. Although we did not examine eye abnormalities, the International Mouse Phenotyping Consortium (IMPC) reports that 13/14 *Myo10*^*tm2/tm2*^ mice had persistent hyaloid vasculature (bilateral in 8/13 cases), 9/14 had fused cornea and lens (7/8 presented in both eyes), 9/14 had corneal opacity (7/9 presented in both eyes), and 3/14 had an abnormal pupil light response. These eye phenotypes are similar to the 11/14 of *Myo10*^*tm1d/tm1d*^, 5/5 of *Myo10*^*tm1a/tm1a*^, and 5/5 of *Myo10*^*m1J/m1J*^ mice that had persistent hyaloid vasculature that was pigmented and bilateral^26^. Other phenotypes associated with the *Myo10*^*tm2(KOMP)Wtsi*^ allele including fused phalanges are described on the IMPC website (http://www.mousephenotype.org/data/genes/MGI:107716).

**Table 1:** Comparison of *Myo10*^*tm2/tm2*^ with *Myo10*^*tm1d/tm1d*^, *Myo10*^*tm1a/tm1a*^*, and Myo10*^*m1J/m1J*^.

Traits	*Myo10* ^*tm1d/tm1d*^ *and Myo10* ^*tm1a/tm1a*^ Data from Heimsath *et al*.^26^	*Myo10* ^*m1J/m1J*^ Data from Heimsath *et al*.^26^	*Myo10* ^*tm2/tm2*^ Data from this report and the IMPC (http://www.mousephenotype.org/data/genes/MGI:107716)
Protein Expression	(−) full-length Myo10 (−) headless Myo10	(−) full-length Myo10 (−) headless Myo10	(−) full-length Myo10 (+) headless Myo10
Birth Rate (Homozygous/wild type)	*tm1d*; 40% *tm1a*; 45%	29%	50%
White Belly Spot in Homozygote	100%	100%	100%
Syndactyly in Homozygote	50%	43%	72%
Kinked Tail in Homozygote	*tm1d*; 75% *tm1a*; 50%	0%	1.6%
Eye Abnormalities	11/14 *Myo10*^*tm1d/tm1d*^ mice and 5/5 *Myo10*^*tm1a/tm1a*^ mice had persistent hyaloid vasculature that was pigmented and bilateral	5/5 *Myo10*^*m1J/m1J*^ mice had persistent hyaloid vasculature that was pigmented and bilateral	13/14 *Myo10*^*tm2/tm2*^ mice had persistent hyaloid vasculature (bilateral in 8/13 cases), 9/14 had fused cornea and lens (7/8 presented in both eyes), 9/14 had corneal opacity (7/9 presented in both eyes), and 3/14 had an abnormal pupil light response

### Myo10kd in the mouse melanoblast cell line Melb-a reduced the number of LPs and migration

**Figure 3:**
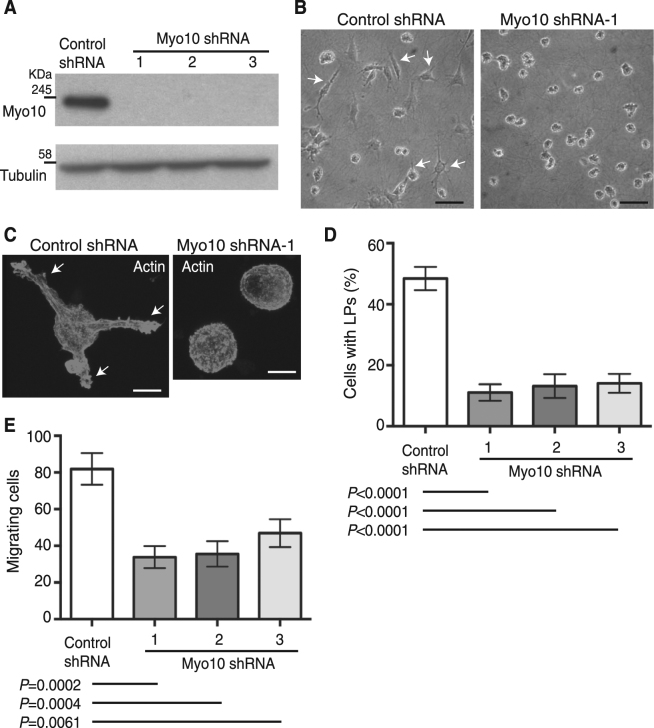
Myo10kd in the mouse melanoblast cell line Melb-a reduced the number of cells with LPs and cell migration. (**A**) Immunoblotting of Myo10 in cell lysates from Melb-a cells transfected with control or a Myo10-specific shRNA. Tubulin served as a loading control. Whole blots are shown in Supplementary Fig. S2. (**B**) Representative phenotype of live Melb-a cells cultured on thick collagen matrices. Control cells show LPs, elongated structures protruding in the direction of cell migration (left panel, arrows), whereas Myo10kd reduced the number of cells with LPs (right panel). Scale bar is 50 μm. (**C**) Melb-a cells in B were fixed and stained for F-actin with fluorescent phalloidin. Control cells have long F-actin-rich protrusions (left panel; arrows), but Myo10 kd cells do not (right panel). Scale bar is 10 μm. (**D**) The number of cells with LPs was counted manually and divided by the total number of cells. The percentage of Myo10kd cells with LPs was 11.1% ± 2.7 (mean ± SEM, 4 different random fields × 3 experiments, *n* = 12, shRNA-1), 12.2% ± 3.9 (*n* = 12, shRNA-2), and 14.1% ± 3.1 (*n* = 12, shRNA-3), which were significantly lower (*P* < 0.0001) than 48.4% ± 3.8 (*n* = 12) in control shRNA cells. (E) Quantification of migrating cells in transwell migration assays. The average number of Myo10kd cells that migrated onto the lower surface of the filter was 33.8 ± 6.0 (mean ± SEM, 4 different random fields × 3 experiments, *n* = 12, shRNA-1), 35.6 ± 7.0 (*n* = 12, shRNA-2), and 46.9 ± 7.6 (*n* = 12, shRNA-3), which were significantly fewer (*P* = 0.0002; shRNA-1, *P* = 0.0004; shRNA-2 and *P* = 0.0061; shRNA-3) than 81.9 ± 8.6 (*n* = 12) in control shRNA cells.

The white belly spot phenotype is caused by reduced migration of melanoblasts during embryonic development, and proper formation of LPs (also referred to as long pseudopods) is the driving force of melanoblast migration *in vivo*^43^. To determine the functional consequences of *MYO10* loss in melanoblasts, we used shRNA to silence *MYO10* in Melb-a cells. The Melb-a cell line was established from mouse melanoblasts, and Melb-a cells retain the character of melanoblasts versus that of mature melanocytes or melanoma cells^44^. Myo10kd was confirmed by immunoblotting of cell lysates from control or Myo10-specific shRNA transfected Melb-a cell lines (Fig. 3A). Images of the whole immunoblots are shown in Supplementary Fig. S2. Myo10kd did not disturb the rate of cell proliferation on culture dishes (not shown).

First, to investigate the effect of Myo10kd on the cellular phenotype of melanoblasts, cells were cultured on thick collagen matrices, and live-cell images were captured. The number of cells with LPs (Fig. 3B, arrows) was 77% lower in Myo10 shRNA-1 cells, 73% lower in Myo10 shRNA-2 cells, and 71% lower in Myo10 shRNA-3 cells vs. cells transfected with scrambled shRNA (Fig. 3B,D). To investigate the F-actin structure in these cells, Melb-a cells were fixed and stained with fluorescent phalloidin (Fig. 3C). In control cells, F-actin accumulated in LPs (Fig. 3C, arrows), but in Myo10kd cells, F-actin was diffusely localized on the surface and in fine spikes, and no LPs were observed. These results indicate that Myo10 is important for induction of LPs in melanoblasts. Next, we performed transwell migration assays and found that the number of cells successfully migrating through the membrane pores was 59% lower in Myo10 shRNA-1 cells, 57% lower in Myo10 shRNA-2 cells, and 43% lower in Myo10 shRNA-3 cells than in cells transfected with scrambled shRNA (Fig. 3E). These results are consistent with a previous report showing that Myo10kd inhibits transwell migration of breast cancer cells^27^. These results suggest a link between LP formation and migration of melanoblasts and that Myo10 may play important roles in these processes.

### Loss of Myo10 delayed onset and development of melanoma

We speculate that Myo10 plays an important role not only in melanoblast migration but also in melanoma metastasis. Several key genetic lesions governing human melanoma initiation and progression have been identified. Among them, the earliest and most common is a point mutation (T1779A) in the *Braf* proto-oncogene, which is detected in ~65% of affected individuals^45^. *Braf*^*T1799A*^ encodes Braf^V600E^ (BrafCA), a constitutively active serine kinase that elicits sustained activation of the Braf/MEK1/2/ERK1/2 MAP kinase pathway. BrafCA stimulates constitutive cell signaling, growth factor-independent proliferation, and transformation of immortalized melanocytes, allowing these cells to grow as tumors in nude mice. Although it contributes to the aberrant pathophysiological characteristics of the melanoma cell, oncogenic activation of *Braf* in melanocytes is insufficient for full malignant conversion. Hence, progression to malignant melanoma is invariably accompanied by silencing of one or more tumor suppressor genes, most commonly *Pten* or *CDKN2A*^46–48^. The combination of *Braf*^*T1799A*^ and *Pten* silencing is common in human melanoma (~20%)^49^, and genetic evidence for *Braf* and *Pten* cooperation in this disease has been obtained by mouse experiments. Upon induction of BrafCA in melanocyte-specific expression, mice develop benign melanocytic hyperplastic growths that fail to progress to melanoma^50^. By contrast, expression of BrafCA combined with Pten tumor suppressor gene silencing elicits development of melanoma and remote metastasis with 100% penetrance^50^.

**Figure 4:**
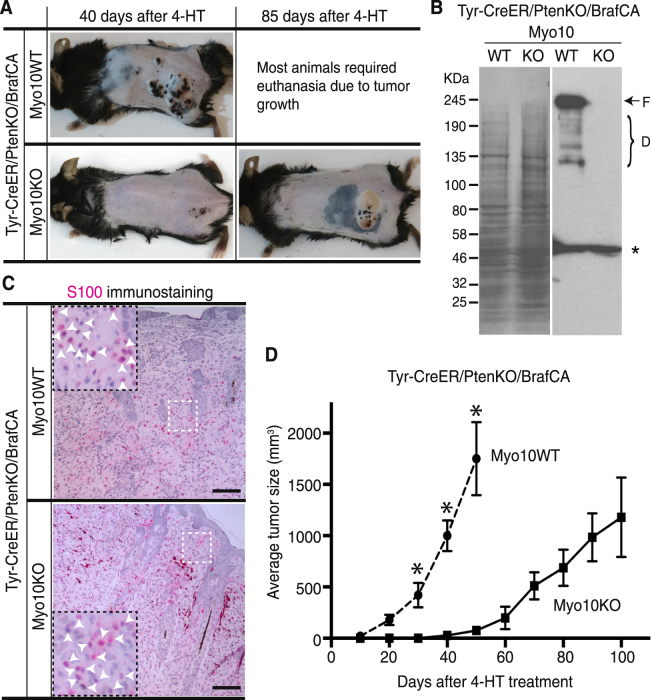
Loss of Myo10 delayed the onset and development of melanoma. (**A**) Representative images of induced melanoma in Myo10WT and Myo10KO mice under the genetic background of Tyr-CreER/PtenKO/BrafCA at the indicated number of days after local 4-HT administration. (**B**) Immunoblotting of tumor samples from WT (+/+) and Myo10 KO (*tm2/tm2*) mice with Tyr-CreER/PtenKO/BrafCA genotype. No expression of full-length Myo10 (F) was observed in the KO tumor. The bands labeled with D are breakdown products; the band labeled with an asterisk is non-specific. Membrane staining with CBB (left panel) after probing showed similar protein loading. (**C**) Detection of S100-positive cells in tumors 40 days (Myo10 WT) and 85 days (Myo10KO) after 4-HT induction. Tumor cells were S100-positive in both genotypes. Inserts are 2.5 × enlargements of boxed areas. Arrows point to S100-positive cells. Scale bar is 200 μm. (**D**) Tumor growth curves for Tyr-CreER/PtenKO/BrafCA/Myo10WT (*n* = 10) and Tyr-CreER/PtenKO/BrafCA/Myo10KO (*n* = 11) genotypes. Average tumor size (mm^3^, mean ± SEM; *y* axis) was plotted as a function of the number of days after 4-HT treatment (*x* axis). Average tumor size in Myo10KO mice was 6 ± 5 mm^3^, which was significantly smaller (*P* = 0.0045) than the average tumor size of 419 ± 122 mm^3^ in Myo10WT mice at day 30; 28 ± 20 mm^3^ in KO and 1045 ± 157 mm^3^ in WT (*P* < 0.0001) at day 40; and 75 ± 45 mm^3^ in KO and 1750 ± 358 mm^3^ in WT (*P* = 0.0003) at day 50.

We therefore crossed Myo10KO mice with a genetically modified model of metastatic malignant melanoma, Tyr-CreER/PtenKO/BrafCA mice^50^, to assess whether Myo10 is important for primary melanoma development and/or metastasis. Local administration of 4-hydroxytamoxifen (4-HT) to adult animals allowed tumor formation to be monitored at the point of application. Tyr-CreER/PtenKO/BrafCA/Myo10WT mice developed primary melanomas with a similar penetrance and latency to that previously described^50^ (Fig. 4A). To investigate the expression of Myo10 in these induced melanoma, we performed immunoblot analysis of whole tumor lysates and found that only the WT mice expressed full-length Myo10, and neither WT nor mutant mice expressed headless Myo10 (Fig. 4B). Tumor cells in both genotypes were positive for S100, a marker with excellent sensitivity for pigmented and amelanotic melanoma (Fig. 4C). Onset of tumors was delayed by Myo10 inactivation, and the average tumor volumes were also markedly reduced from day 30 in Tyr-CreER/PtenKO/BrafCA/Myo10KO mice (Fig. 4A,D). These findings provide the first genetic evidence for the involvement of *MYO10* in melanoma development as reflected by the longer disease latency and reduced tumor size in the Myo10KO mice.

### Genetic inactivation of *MYO10* inhibited melanoma metastasis and increased survival

**Figure 5:**
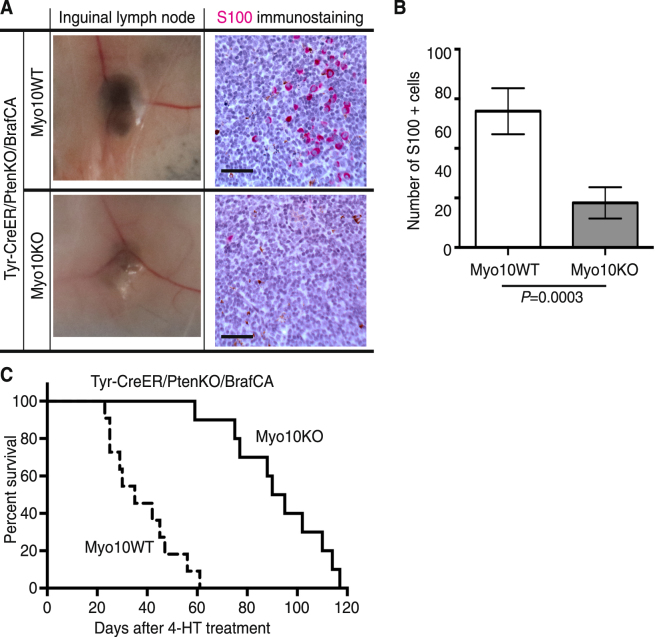
Genetic inactivation of *MYO10* inhibited melanoma metastasis and increased survival. (**A**) Representative images of inguinal lymph nodes and immunohistochemistry of S100 in Tyr-CreER/PtenKO/BrafCA/Myo10KO and Tyr-CreER/PtenKO/BrafCA/Myo10WT mice. Scale bar is 100 μm. (**B**) Quantification of S100-positive melanocytes in regional lymph nodes. The average number of S100-positive cells in lymph nodes in Tyr-CreER/PtenKO/BrafCA/Myo10KO mice was 18.0 ± 6.3 (mean ± SEM, 6 random fields × 3 slices × 10 mice, *n* = 180), which was significantly fewer (*P* = 0.0003) than 55.9 ± 8.5 (*n* = 198) in lymph nodes from Tyr-CreER/PtenKO/BrafCA/Myo10WT mice. (**C**) Kaplan-Meier survival curve of mice with the indicated genotypes, Myo10WT (*n* = 10) or KO (*n* = 11) under the genetic background of Tyr-CreER/PtenKO/BrafCA following local administration of 4-HT. Log-rank (Mantel-Cox) test of survival plots revealed a significant difference (*P* < 0.0001) between the Myo10 WT and KO genotypes.

To study Myo10 function in melanoma metastasis, we performed necropsy of Tyr-CreER/PtenKO/BrafCA/Myo10KO and Tyr-CreER/PtenKO/BrafCA/Myo10WT mice when tumor volumes reached ~1,000 cm^3^. Mice that received 4-HT on the left side of the dorsal skin showed evidence that the melanoma had spread into the left inguinal lymph node based on the swelling and pigmented appearance observed in Tyr-CreER/PtenKO/BrafCA/Myo10WT mice (Fig. 5A). The number of S100-positive cells in lymph nodes was 68% fewer in the Tyr-CreER/PtenKO/BrafCA/Myo10KO animals than in the Tyr-CreER/PtenKO/BrafCA/Myo10WT animals (Fig. 5A,B). The medial survival time of Tyr-CreER/PtenKO/BrafCA mice was extended ~264% (from 35.0 to 92.5 days) by inactivation of *MYO10* (Fig. 5C). Log-rank (Mantel-Cox) test of survival plots revealed a significant difference between the Myo10WT and KO genotypes under the genetic background of Tyr-CreER/PtenKO/BrafCA, evidence of Myo10’s role in melanoma progression.

### Myo10kd in B16F1 cells reduced LP formation in 3D collagen matrices and lung colonization after intravenous injection

**Figure 6:**
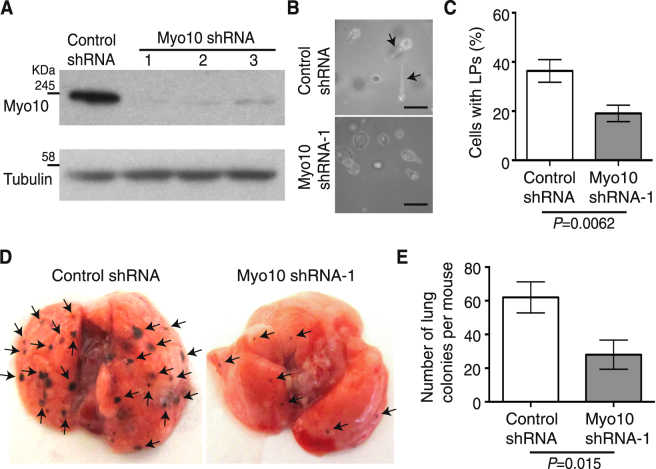
Myo10kd in B16F1 cells reduced the number of cells with LPs in 3D collagen matrices and lung colonization after intravenous injection. (**A**) Immunoblots of Myo10 in cell lysates from B16F1 control cells or B16F1 cells transfected with Myo10-specific shRNA. Tubulin served as a loading control. Whole blots are shown in Supplementary Fig. S3. (**B**) Representative phenotype of cells cultured in 3D collagen matrices. Control shRNA cells showed LPs (upper panel, arrows), whereas Myo10kd (Myo10 shRNA-1, lower panel) inhibited protrusion formation. Scale bar is 50 μm. (**C**) The average percentage of cells with LPs in Myo10 shRNA-1 cells was 19.1% ± 3.4 (mean ± SEM, 4 different random fields × 3 experiments, *n* = 12), which was significantly lower (*P* = 0.0062) than 36.3% ± 4.6 (*n* = 12) in control shRNA cells. (**D**) Representative images of lungs isolated 2 weeks after intravenous injection of B16F1 control or Myo10kd cells. Arrows indicate colonies of B16F1 cells. (**E**) Quantification of the number of lung colonies detected per animal. The average number of lung colonies in mice injected with Myo10 shRNA-1 cells was 28.0 ± 8.6 (mean ± SEM, *n* = 10), which was significantly fewer (*P* = 0.015) than 62.0 ± 9.3 (*n* = 10) found in lungs of mice injected with control shRNA cells.

To determine whether Myo10KO in melanoma cells is responsible for the observed inhibition of metastasis in mice, we employed B16F1 mouse melanoma cells in lung colony formation assays. The migration of B16F1cells in 3D collagen lattices is accompanied by dynamic changes in the morphology of a prominent actin-rich protrusion at the leading edge^51^. First, we established B16F1 Myo10kd cell lines and confirmed that the knockdown effect was >90% as compared to B16F1 cells expressing control shRNA (Fig. 6A). Images of the whole immunoblots are shown in Supplementary Fig. S3. Myo10kd did not change the proliferation rate of B16F1 cells cultured in complete medium on culture dishes (data not shown). The number of cells with LPs (Fig. 6B, arrows) cultured in collagen matrix was 47% fewer in Myo10 shRNA-1 cells than in control shRNA cells (Fig. 6B,C). Next, we analyzed lung colony formation after intravenous injection of control shRNA and Myo10 shRNA-1 cells into the tail vein of mice and found that the number of lung colonies after two weeks was ~55% fewer in Myo10kd vs. control cells (Fig. 6D,E). These results suggest that attenuation of LP formation by Myo10kd inhibited the extravasation of B16F1 cells and colony formation in the lung and indicate that the observed inhibition of melanoma metastasis in the previous experiments was due to reduced Myo10 expression in melanoma.

### Myo10 localized at the tips of protrusions in 3D migrating B16F1 cells, and Myo10kd reduced VASP localization at these sites

**Figure 7:**
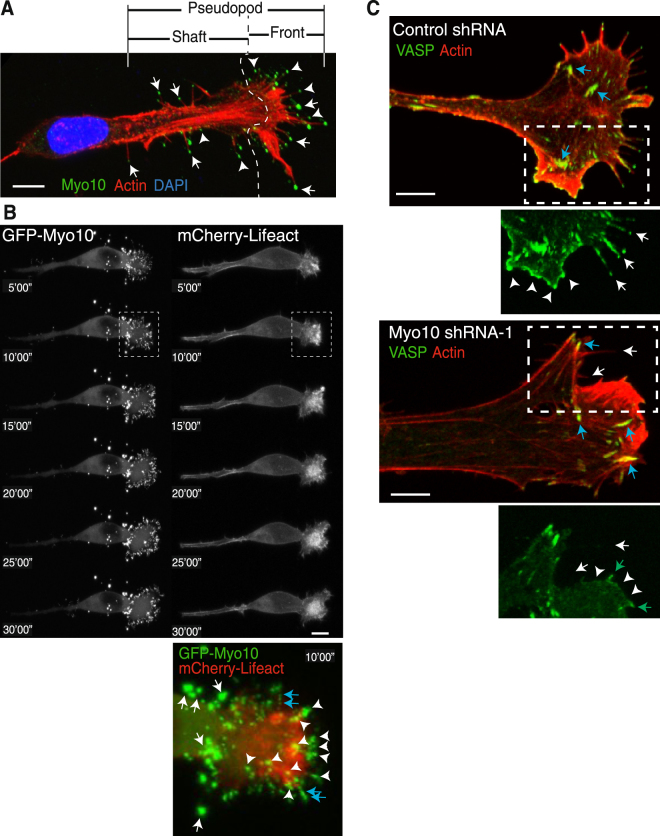
Myo10 localized at the front of pseudopods in 3D migrating cells, and Myo10kd reduced VASP localization at these sites. (**A**) An image showing the typical phenotype of B16F1 cells that migrate near the bottom of a 3D collagen matrix. Cells were fixed and stained with anti-Myo10 antibody (green), fluorescent phalloidin (red), and DAPI (blue). A broad (~5 μm) and long (>20 μm) pseudopod defines the direction of the migrating cell and consisted of two parts: a shaft (left of the dotted line) and a front (right of the dotted line). Several filopodia (sometimes dozens) extended from the front of the pseudopod. Longer and stable filopodia were filled with F-actin (arrows), but F-actin was difficult to detect in the shorter ones (arrowheads). Myo10 accumulated at the tips of almost all the filopodia. Scale bar is 10 μm. (**B**) A representative time-lapse image of a B16F1 cell that has migrated into the 3D collagen matrix. GFP-Myo10 (left) and mCherry-Lifeact (right) were co-expressed in B16F1 cells and monitored in 5 min intervals over a 30 min period, and movies were generated from these images (Supplementary Movies S1 and S2). The lower panel shows a magnified view (box, 10′00″) of the front of the pseudopod at 10 min. White arrows indicate Myo10 punctae in the shaft of the pseudopod; arrowheads indicate Myo10 at the front of the pseudopod; and blue arrows indicate where Myo10 appears to form a continuous track due to its movement back and forth along actin filaments in filopodia within a single movie frame. (**C**) Knockdown of Myo10 reduced VASP localization at the front of pseudopods. B16F1 cells transfected with control shRNA (upper panels) or Myo10 shRNA-1 (lower panels) were cultured in 3D collagen matrices, and stained with anti-VASP antibody (green) and fluorescent phalloidin (red). Scale bar is 5 μm.

Migrating B16F1 cells in collagen lattices form pseudopods with multiple finger-like termini^51^. The kinetics of the leading edge and the traction of collagen fibers suggested a new perspective on 3D migration of melanoma cells in which the dynamics of filopodia mediate force generation and translocation of the leading edge. At the front of the pseudopod, multiple filopodia transiently bind to and pull on collagen fibrils, causing a reversible change in the position of both the filopodia and fibrils^51^. Thus, we hypothesized that Myo10 localizes at the tips of filopodia projecting from pseudopods and mediates membrane dynamics at the leading edge of migrating melanoma cells. To investigate this possibility, first we localized Myo10 in B16F1 cells cultured in 3D collagen matrices. Figure 7A shows the typical phenotype of a B16F1 cell migrating near the bottom of a 3D collagen matrix. A broad (~5 μm) and long (>20 μm) protrusion, or pseudopod, was observed at the front of the migrating cell and consisted of two parts: a shaft (left of the dashed line, Fig. 7A) and front (right of the dashed line). Several (sometimes dozens) filopodia were observed at the front of the pseudopod. Longer and stable ones were filled with F-actin (Fig. 7A, arrows), but it was difficult to detect F-actin in shorter ones (Fig. 7A, arrow heads). Filopodia existed also at the shaft of the pseudopods. Myo10 was accumulated at the tips of almost all of the filopodia.

Next, we transfected GFP-Myo10 and mCherry-Lifeact to monitor Myo10 dynamics and actin accumulation, respectively, in 3D migrating live cells. GFP signals were diffuse in the cytoplasm and more highly concentrated in puncta, and mCherry signals were also diffuse in the cytoplasm and accumulated in multiple finger-like termini at the leading edge (Fig. 7B, upper panel). In a magnified view of a pseudopod excerpted from the area in the time lapse images denoted by a dotted box (Fig. 7B, lower panel), two different types of GFP-positive puncta were detected: small ones (arrowheads) that localized at the front of the pseudopod, and larger ones (arrows) that localized at the shaft of the pseudopod. The smaller puncta located mainly at the tips of the filopodia projecting from the front of the pseudopod, and these filopodia dynamically elongated and retracted (Supplementary Movies S1 and S2) so that several of them appeared to form a continuous line because they were captured several times in one time frame (lower panel, blue arrows). Bigger puncta behind the leading edge were stable and did not change location during the recorded period. These results suggest that Myo10 induces filopodia projecting from pseudopods and mediates dynamics at the front of pseudopods in 3D migrating melanoma cells.

In addition to actin, finger-like protrusions at the leading edge contained foci of VASP^51^. VASP, which exhibits anti-capping activity during actin assembly, is a major inducer of filopodia and lamellipodia^52,53^. We previously reported that Myo10 transports VASP to the tips of filopodia^5^. From these results, we speculated that Myo10 transports VASP to the tips of filopodia protruding from pseudopods in melanoma cells. To study this possibility, VASP localization at the tips of 3D pseudopods was compared between control (upper panels) and Myo10 shRNA-1 (lower panels) transfected B16F1 cells (Fig. 7C). In control cells, VASP localized in foci at the tips and along the shafts of filopodia (arrows) and at the tips of membrane ruffles (arrowheads). On the other hand, pseudopods rarely formed in Myo10kd cells. Fewer filopodia were evident and little or no VASP localized in filopodia (arrow) and ruffles (arrowheads), although some VASP were accumulated in short spikes (green arrows). The localization of VASP in focal adhesions was the same as in control cells (blue arrows). These results are consistent with a model in which Myo10 transports VASP to the leading edge of 3D migrating melanoma cells, which accelerates actin polymerization required for invasion.

### *MYO10* expression increased in human melanoma and decreased in melanoma with *TP53* mutation

**Figure 8:**
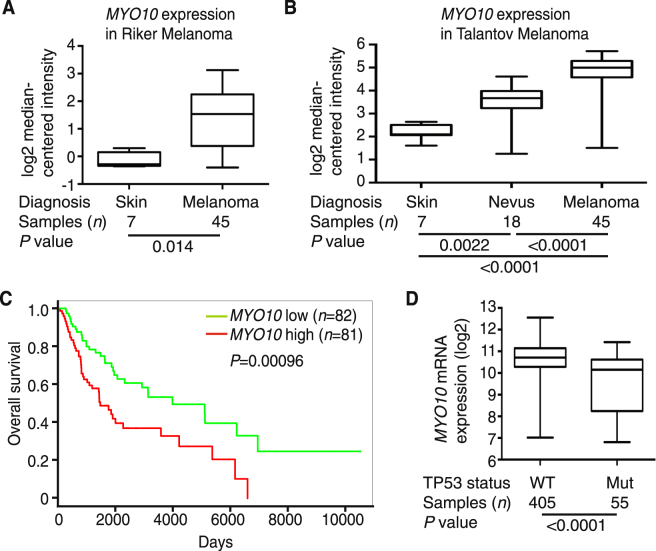
Increased *MYO10* expression in human melanoma and relationship with *TP53* mutation. Oncomine analysis of *MYO10* expression in malignant melanoma compared with normal tissues. (**A**) Box-and-whisker plots show that *MYO10* mRNA expression was significantly higher (*P* = 0.014) in melanoma (*n* = 45) than in normal skin (*n* = 7) in the Riker melanoma data set GSE7553. (**B**) *MYO10* mRNA expression was significantly higher in nevi (*n* = 18) than in normal skin (*n* = 7, *P* = 0.0022), in melanoma (*n* = 45) than in nevi (P < 0.001), and in melanoma than in normal skin (P < 0.001) in the Talantov melanoma data set GSE3189. (**C**) *MYO10* expression and survival (Kaplan-Meier curves) were analyzed in 163 patients with malignant melanoma (TCGA-SKCM). Log-rank test *P* value, *P* = 0.00096. (**D**) *MYO10* mRNA expression was significantly lower (*P* < 0.0001) in samples with *TP53* mutants (Mut, *n* = 55) than in wild type (WT, *n* = 405) in the skin cutaneous melanoma data set (TCGA, Provisional) downloaded from the cBioPortal database.

To investigate the role of Myo10 in human melanoma, we first analyzed the expression of *MYO10* mRNA in human skin, benign nevi, and melanoma samples by *in silico* analysis of Oncomine datasets. *MYO10* mRNA was significantly higher in melanoma than in normal skin in the Riker melanoma data set (Fig. 8A) and in the Talantov melanoma data set (Fig. 8B). Also, *MYO10* mRNA expression was significantly higher in melanoma than in nevi in the Talantov melanoma data set (Fig. 8B). These results suggest that *MYO10* mRNA expression is higher in human melanoma than in normal skin and nevi.

Next, we used the PROGgeneV2 prognostic biomarker identification tool^54^ to study the implications of *MYO10* gene expression on overall survival of 163 melanoma patients in The Cancer Genome Atlas-Skin Cutaneous Melanoma (TCGA-SKCM) data sets. Using median gene expression values as bifurcation points, Cox proportional hazards analyses showed that elevated expression of the *MYO10* gene was associated with inferior survival outcomes (Fig. 8C). Kaplan-Meier plots indicated significant segregation in survival outcomes for patients with high vs. low *MYO10* expression (hazard ratio, HR = 1.43; confidence interval, CI, 1.16–1.77; *P* = 0.00096).

A recent study showed that *MYO10* mRNA expression was significantly higher in breast tumors with mutant p53^28^. To determine if this same correlation exists for melanoma, we compared *MYO10* mRNA expression relative to the *TP53* status in melanoma samples. Interestingly, *MYO10* mRNA expression was significantly lower in samples expressing *TP53* mutations vs. wild-type *TP53*, the opposite of that observed in breast cancers (Fig. 8D). In prostate cancer cell lines, there was no link between Myo10 overexpression and expression of mutant p53^32^. These results suggest that regulation of *MYO10* mRNA expression is different among melanoma, breast cancer, and prostate cancer.

### Myo10 expression is higher in melanoma than in nevi

**Figure 9:**
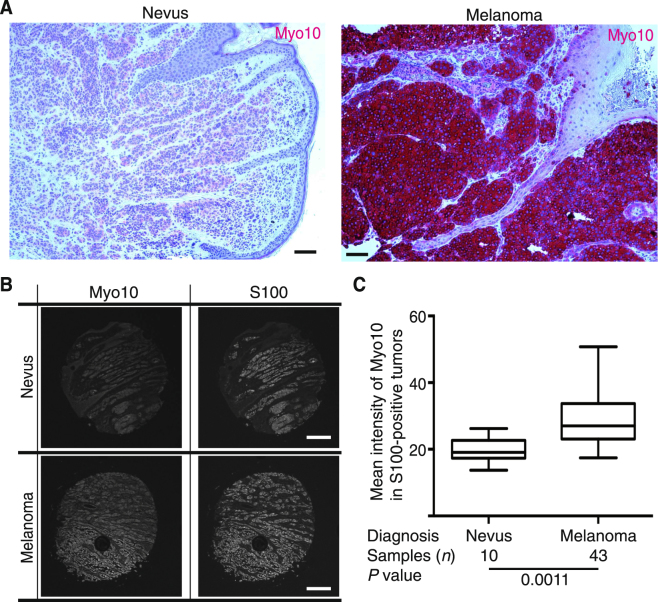
Increased Myo10 expression in human melanoma. (**A**) Examples of Myo10 immunostaining in human nevus (left) and melanoma (right) samples. Scale bar is 200 μm. (**B**) Expression of Myo10 was studied in a tissue microarray consisting of primary and metastatic melanomas and nevi. Examples of Myo10 and S100 staining in melanoma and nevus are shown. (**C**) Box-and-whiskers plots demonstrated differences in Myo10 expression in the nevi (*n* = 10) and melanoma (*n* = 43) samples. Myo10 expression was significantly higher in melanoma lesions than in nevi (*P* = 0.0011).

To confirm Myo10 protein expression in human melanoma, we stained randomly selected tissue samples of diagnosed benign nevi (*n* = 5) and malignant melanoma (*n* = 9) archived in the SkinPath Laboratory of Boston University School of Medicine. Figure 9A shows examples of low Myo10 expression in nevus (left panel) and high expression in melanoma (right panel) by immunostaining. There was a tendency for higher Myo10 expression in melanoma (*n* = 5, high; 3, medium; and 1, low) vs. nevi (*n* = 2, medium; and 3, low), but the sample number was not large enough to determine whether there was a statistical difference in expression levels between the two groups. Thus, we used a tissue microarray (TMA; ME1004e; US Biomax, Inc.) consisting of both melanomas and nevi on one slide allowing for staining of all cores at the same time (Fig. 9B). We performed immunohistochemistry on the TMA with anti-Myo10 antibody and anti-S100 antibody, and the intensity of Myo10 staining was captured only in S100-positive regions and calculated per pixel. Box-and-whiskers plots demonstrated that Myo10 expression was significantly higher in melanoma lesions than in nevi, consistent with the data of *MYO10* mRNA expression (Fig. 8B). These results suggest that Myo10 expression level correlates with melanocytic neoplastic progression in humans and overall survival of melanoma patients.

## Discussion

### Myo10KO phenotype

We show that the Myo10KO mice used in this study, *Myo10*^*tm2/tm2*^, express headless Myo10 in brain, but do not express full-length Myo10 in brain or kidney. These results are consistent with the allele structure of *Myo10*^*tm2(KOMP)Wtsi*^, which has intact alternative transcription start sites and first exons specific for *headless Myo10*. After this study was submitted, a new article describing Myo10KO mice generated with a different KO strategy was published^26^. In that study, the *Myo10*^*tm1a*^ and *Myo10*^*tm1d*^ mutations target exon 27, and the *Myo10*^*m1J*^ mutation deletes 8 bp in exon 25. None of those three KO mice (*Myo10*^*tm1a/tm1a*^, *Myo10*^*tm1d/tm1d*^, and *Myo10*^*m1J/m1J*^) expresses either full-length *or* headless Myo10 in brain. Interestingly, we found that the phenotype of the total KO mouse (i.e., knockout of both *full-length Myo10* and *headless Myo10*; *Myo10*^*tm1a/tm1a*^, *Myo10*^*tm1d/tm1d*^, and *Myo10*^*m1J/m1J*^) and the *full-length Myo10* only KO mouse (*Myo10*^*tm2/tm2*^) is almost the same. This conclusion is reasonable because most tissues express only full-length Myo10, whereas neural stem cells, neurons, and astrocytes express both full-length and headless Myo10^22,42^. Thus, the difference between the total KO and the full-length only Myo10 KO may be restricted to these neuronal cells. One idea is that headless Myo10 is a negative regulator of full-length Myo10^22^, but whether headless Myo10 plays other functions is largely unknown. It is plausible that new insight into the function of headless Myo10 *in vivo* will result from comparion studies of neuronal function in *total Myo10* KO mice vs. *full-length Myo10 only* KO mice.

### Myo10 function in melanoblast migration

We found that Myo10KO induces white belly spots in mice and that Myo10kd in melanoblast cells reduces LP formation, evidence that Myo10 controls melanoblast migration by mediating LP formation. Rac1 positively regulates the frequency of initiation of LPs in melanoblasts, which control migration speed and directional plasticity^43^; and Rac1KO in melanocytes results in a white belly spot phenotype. Loss of the Rac exchange factor P-Rex1 also causes white belly spots and inhibition of melanoma metastasis^55^. Thus, the PIP_3_/P-Rex1/Rac1/Scar/WAVE/Arp2/3 signaling pathway is speculated to be a major pathway for LP-driven migration of the melanocyte lineage^55^. A recent study demonstrates that Cdc42 null mice display a severe loss of pigmentation, a white patch covering half to most of the underside, and that melanoblasts show cell-cycle progression, migration, and cytokinesis defects^56^. Cdc42KO melanoblasts migrate slowly and inefficiently in the epidermis with bulky elongated LPs. These results suggest that proper length and dynamics of LPs are important for normal migration of melanoblasts *in vivo*. Recent findings suggest that PIP_3_ binding is important for the localization of Myo10 at the plasma membrane in order to induce filopodia formation^10^. Also, Myo10 is thought to exist as a monomer whose tail is folded back onto the head/neck region in an auto-inhibited “off” state^4^. Binding to PIP_3_ induces unfolding of the molecule and promotes dimerization of Myo10 to form an active motor able to promote actin fiber convergence at the cell periphery, which initiates filopodia formation^57^. Thus, Myo10 is possibly involved in the PIP_3_-mediated signaling pathway that induces LPs in melanoblasts.

Melanoblasts, which derive from neural crest cells (NCCs), are precursors to melanocytes. NCCs first differentiate into founder melanoblasts in the trunk region on mouse embryonic day 8.5–9.5 (E8.5-E9.5). These founder melanoblasts proliferate in a wedge-shaped area (migration staging area) in the ectoderm, the neural tube and the somites. On E10.5 these melanoblast precursors, which are still proliferating, begin to migrate in between the somites and the ectoderm along a rostro-caudal temporal gradient. Some of the migrating melanoblasts begin to cross the basement membrane (BM) separating the dermis from the epidermis on E11.5. The remaining cells continue to migrate dorsolaterally in the dermis until E15.5 while continuing to cross the BM^58^. Loss of Myo10 reduces the number of filopodia and junction formation in cultured kidney epithelial cells^21^. There are no available data about Myo10 function in junction formation in skin keratinocytes, but it is possible that melanoblast migration is regulated by the surrounding epidermal cells.

### Myo10 in melanoma development and metastasis

In the current study we found that Myo10KO significantly reduced melanoma development and metastasis in Tyr-CreER/PtenKO/BrafCA mice. These results suggest that Myo10KO attenuates the PtenKO and/or BrafCA effects in melanocyte neoplastic progression. PtenKO increases the concentration of PIP_3_ in melanocytes, and PIP_3_ binding activates Myo10 in one of two ways: (1) by targeting Myo10 to the plasma membrane, and (2) by inducing a conformational change in the Myo10 molecule itself as discussed above. Thus, if the main target of the Pten/PIP_3_ signaling pathway is to activate Myo10 so that it induces pseudopod formation required for melanoma induction/metastasis, Myo10KO could diminish the PtenKO phenotype. Using “Matrigel on top” 3D culture models, the formation of actin-rich filopodial-like protrusions (FLPs) was identified as critical for extravasation of melanoma cells^59^. When mice are inoculated with aggressive cancer cells, abundant formation of FLPs is found at the inoculation site and in lung metastases. The number of FLPs was decreased by blocking either the Rif/mDia2 or ILK/β-parvin/cofilin signaling pathways resulting in a reduction in proliferation and metastasis. Pseudopods are thought to be similar to FLPs structurally and functionally and required not only for metastatic outgrowth, but also for primary tumor formation by interacting with the surrounding extracellular matrix^59^. Myo10 transports integrin-β to the tips of filopodia for their stability and elongation^7^. FLPs contain integrin-β1 for outside-in signaling transduction for proliferation. Thus, reducing the formation of actin-based protrusions by Myo10KO may decrease the proliferation signaling induced by BrafCA mutation.

### Melanoma cell migration *in vivo* and in 3D culture models

The mechanism of melanoma migration/metastasis *in vivo* is still controversial, and cancer cells use different modes of migration, which are influenced by a number of intrinsic and extrinsic cues^60^. Using a combination of single and multiphoton confocal microscopy, migration of melanoma cells expressing a fluorescent marker can be monitored *in vivo*^61^. Even in an aggressive mouse line (B16F2) only 6–7% of the cells are motile *in vivo*, and most motile cells use an amoeboid-like single-cell mode of motility. It is possible that these amoeboid-like cells have fine protrusions such as FLPs or pseudopods, but that they are not detected because they are below the limit of resolution.

To overcome the limitation of *in vivo* observation, we used a 3D collagen culture system to monitor the morphology of live cells during migration. In this system, migrating mouse and human melanoma cell lines both showed similar morphodynamics of the finger-like filopodia at the front of the cells^51^. Our results suggest that Myo10 binds and transports the actin-polymerization machinery (including VASP) to the tips of pseudopods, where actin assembly occurs. It is possible that in addition to Myo10 other factors are also important for VASP targeting to the tips of pseudopods. Actin filaments then bundle into thin protrusions to form filopodia that push out the cell membrane in the direction of migration^57^. In 3D migration, integrins also play an important role by attaching the cell to the surrounding extracellular matrix (ECM). Activated β1 integrin localizes at every contact point the cell makes along its length with the fibers comprising the ECM^62^, and recycling of α5β1 integrin enhances the invasive migration of cancer cells in the three-dimensional ECM, which is driven by filopodial structures, which resemble pseudopods with filopodia at their front^63^. Myo10 binds (through its FERM domain) and transports β1 integrin to the tip of filopodia, which may serve as adhesive structures that support filopodial extension^7,64^. Induced filopodia in cells expressing the FERM-deletion mutant of full-length Myo10 do not stably extend^65,66^. These results suggest the possibility that Myo10 transports integrins into the leading front of pseudopods, which enable cells to adhere to the ECM fibers and move forward. Myo10 may control a balance between adhesion to ECM fibers driven by integrins and actin polymerization driven by VASP at the leading front of pseudopods.

For primary melanoma cells to establish remote metastases, they must invade other tissues/organs by breaching barriers such as the BM and stromal and/or parenchymal regions of target organs. Myo10 is expressed ubiquitously in almost all organs so these barriers may be altered in Myo10KO mice. Myo10kd in B16F1 cells reduced lung colonization after tail vein injection indicating that the effect is autonomous to melanoma cells, although it is still possible that Myo10KO in another organ may positively or negatively contribute to melanoma metastasis in Myo10KO mice.

### Myo10 expression in human melanoma

Recent studies suggest that Myo10 has important functions in various types of cancer, e.g., breast cancer^27,28,31^, lung adenocarcinoma^29^, non-small cell lung cancer^30^, and prostate cancer^32^. In this study, we present the first genetic evidence that Myo10KO delays the onset and development of melanoma and reduces its metastasis *in vivo*. In human melanoma, increased expression of Myo10 decreases patient survival, which is the same effect observed in breast cancer. It is not clear whether Myo10 mediates the same mechanism in the progression of these types of cancer, but our findings in melanoma may provide a new direction to tackle other types of cancer.

In summary, we propose a Myo10-mediated LP induction model in melanoblast migration and melanoma induction and metastasis. Melanoblast and melanoma share a similar mechanism for migration and proliferation, although they are not completely the same. If the majority of intrinsic and extrinsic cues converge at LP formation, Myo10 may play a pivotal role in these phenotypes. Thus, Myo10 may be a therapeutic target for melanocyte-related diseases.

## Methods

### Mouse models

*Myo10*^*tm2(KOMP)Wtsi*^ (Myo10KO) C57BL/6 N mice were obtained from the Wellcome Trust Sanger Research Institute. The inducible *Tyr::CreER*^*T2*^*;Pten*^*LoxP/LoxP*^*;Braf*^*CA/*+^ melanoma model (Tyr-Cre/PtenKO/BrafCA) has been described before^50^, and we obtained this line from The Jackson Laboratory (Stock No:013590). By crossing these two lines, we generated *Tyr::CreER*^*T2*^*;Pten*^*LoxP/LoxP*^*;Braf*^*CA/*+^*;Myo10*^*−/−*^ (Tyr-Cre/PtenKO/BrafCA/Myo10KO) and *Tyr::CreER*^*T2*^*;Pten*^*LoxP/LoxP*^*;Braf*^*CA/*+^*;Myo10*^+*/*+^ (Tyr-Cre/PtenKO/BrafCA/Myo10WT) mice. All the animal experiments were performed in accordance to the NIH Guide for the Care and Use of Laboratory Animals and were approved by the Institutional Animal Care and Use Committee (IACUC) of Boston University School of Medicine.

### Genotyping

Primer sequences for genotyping *Myo10*^*tm2(KOMP)Wtsi*^ mice were also provided by the Wellcome Trust Sanger Research Institute as follows:

Myo10F: 5′-ATCTGTTTCCCCTTAAGCGAAAAT-3′,

Myo10R: 5′-CTCTGTGGGGCCCAGAGCT-3′,

MutR: 5′-TCGTGGTATCGTTATGCGCC-3′,

LacZF: 5′-ATCACGACGCGCTGTATC-3′,

LacZR: 5′-ACATCGGGCAAATAATATCG-3′.

Genomic DNA samples isolated from tail snips were used as a template for PCR with primer pairs Myo10F/Myo10R, Myo10F/MutR and LacZF/LacZR. PCR with the primer pair Myo10F/Myo10R produced a 400-bp product only in mice that carried at least one copy of the WT allele. PCR with the primer pair Myo10F/MutR resulted in a 295-bp product, and PCR with the primer pair LacZF/LacZR resulted in a 108-bp product in mice that carried at least one copy of the mutant allele. The method for genotyping Tyr-Cre/PtenKO/BrafCA mice has been previously described^50^.

### Activation of the Tyr::CreER^T2^ transgene and mouse survival cohorts

The dorsal hair of three week old mice was shaved and further depilated with depilatory cream (Veet) before topical application to the left side of the dorsal skin of 2 μl 10 mM 4-HT in ethanol (Fisher Scientific). A total of 10 Myo10WT and 11 Myo10KO mice under the background of Tyr-Cre/PtenKO/BrafCA was monitored for up to 120 days for the development of melanoma and signs of metastasis. All mice were checked twice weekly for the development of malignant melanoma, and tumor growth was measured using calipers. Tumor size was estimated using the ellipsoid volume formula: 4/3π(length/2)(width/2)(depth/2). End point criteria were melanomas ≥20 mm in diameter, ulcerating melanomas, cachexia, significant weight loss, or weakness and inactivity. Upon meeting these criteria, mice were euthanized.

### Immunohistochemistry

Tissues were removed from the mice directly after euthanasia. For Trp2 (tyrosinase-related protein 2) staining in hair follicles, excised skin was embedded in OCT compound (Sakura Finetek USA, Inc., Torrence, CA), frozen with dry ice, and stored at −80 °C. Frozen tissues were cut into 8 μm sections, which were then fixed in 4% paraformaldehyde for 10 min and blocked with 5% normal donkey serum in PBS for 30 min. The tissue sections were then incubated with anti-Trp2 antibody (sc-10451, Santa Cruz Biotechnology, Inc., Dallas, TX) diluted with 5% bovine serum albumin in PBS overnight at 4 °C. After washing with PBS, sections were incubated with donkey anti-goat IgG-FITC (sc-2024, Santa Cruz Biotechnology) for 1 h and counterstained with 4′,6-diamidine-2′-phenylindole dihydrochloride (DAPI, Vector Laboratories, Burlingame, CA). Immunofluorescence was observed and photographed on a TCS SP5 confocal microscope (Leica Microsystems, Inc., Buffalo Grove, IL).

S100 proteins are expressed in many types of human cancers^67^, among them, S100A4, S100A9 and S100B are markers with excellent sensitivity for melanoma^68–71^. Mouse monoclonal anti-S100 antibody (4C4.9) was generated with purified bovine brain S100 protein and stained S100 in melanoma and its metastasis^72,73^. For S100 staining in tumors and lymph nodes, tissues were fixed in formalin and embedded in paraffin. Five micrometer-thick sections were cut, de-paraffinized with xylene, and autoclaved for 20 min in sodium citrate buffer (10 mM, pH 6) for antigen retrieval. Staining was performed with reagents from Vector Laboratories. Endogenous alkaline phosphatase was blocked with BLOXALL blocking solution for 10 min. Avidin/Biotin blocking was performed with an Avidin/Biotin blocking kit. M.O.M mouse Ig blocking reagent was used for 1 hr to block endogenous mouse immunoglobulins. After washing with PBS and incubating with M.O.M diluent for 5 min, slides were incubated with anti-S100 antibodies (4C4.9, Thermo Fisher Scientific) in M.O.M diluent for 30 min at room temperature. After washing with PBS, M.O.M biotinylated anti-mouse IgG reagent was applied to the samples for 10 min. Slides were washed with PBS, then incubated with VECTASTAIN ABC-AP reagent for 30 min. After washing with PBS, alkaline phosphatase was localized with ImmPACT Vector Red reagent for 20 min, and slides were counterstained with Vector Hematoxylin QS.

For Myo10 staining in human specimens, formalin fixed and paraffin embedded tissues were cut into 5 μm sections, de-paraffinized, subjected to antigen retrieval, then blocked as described above. After incubation with 2.5% normal horse serum for 30 min, slides were incubated with anti-Myo10 antibody (Sigma HPA024223) diluted with 5% bovine serum albumin in PBS overnight at 4 °C. Slides were then washed with PBS and incubated with ImmPRESS-AP polymer anti-rabbit IgG reagent for 30 min, and counterstained as described above. Standard microscope images were acquired using a Nikon E800 microscope with SPOT camera and software version 4.6 (Diagnostic Instruments Inc., Sterling Heights, MI).

For tissue microarray analysis, a tissue microarray consisting of melanoma and nevi cohorts was obtained from US Biomax, Inc. (ME1004e). The TMA slide was de-paraffinized and antigen was unmasked as described above. After incubation with blocking buffer (10% goat serum, 5% BSA in PBS) for 30 min, anti-Myo10 and anti-S100 antibodies diluted in blocking buffer were applied to the slide overnight at 4 °C. After washing with PBS, slides were incubated with Alexa Fluor 488 goat anti-rabbit IgG and Alexa Fluor 568 goat anti-mouse IgG (Thermo Fisher Scientific) in 5% BSA in PBS for 1 h. Alexa Fluor 488 and 568 fluorescence were captured on the TCS SP5 confocal microscope using sequential acquisition to give separate image files for each. A scan speed of 400 Hz and 4-frame averaging were used, and gain and offset were adjusted to give sub-saturating fluorescence intensity with optimal signal-to-noise ratio. Once the settings were determined, they were used to capture all the cores of the array. Image analysis was performed with ImageJ software. TMA cores with less than 5% tumor area per spot were not included in quantitative analysis. Using ImageJ, binary image masks of Myo10 and S100 were created by setting the lower threshold level at 35 and the upper level at 255. S100-positive staining was used to define regions of interest (ROI) for analysis. The ROI was overlaid onto the Myo10 image, the integrated density in this region was measured, and the total integrated density was divided by the total area of the ROI. This value was considered as the mean intensity of Myo10 in S100-positive tumors.

### Quantification of lymph node metastasis

Metastasis from a primary tumor to the lymph nodes was observed when the volume of the primary tumor reached ~1,000 mm^3^, restricting drainage of the lymph nodes (left inguinal). Fixed and embedded lymph nodes were sliced at the middle of the tissue. All the sections were stained with S100 antibody as described above and scanned manually at a magnification of 20× using the Nikon E800 microscope with SPOT camera and software version 4.6 (Diagnostic Instruments Inc.). S100-positive cells were counted in six random fields in each of three slices per mouse.

### Cell culture, transfection, and capture of live-cell images

Melb-a melanoblasts were kindly provided by Dr. Dorothy C. Bennett (St. George’s University of London, London, UK)^31^, and grown in RPMI supplemented with 10% fetal bovine serum (FBS) (Atlanta Biologicals, Flowery Branch, GA), 2 mM glutamine, 100 international units/ml penicillin, 100 μg/ml streptomycin (=complete medium; all components from Life Technologies, Thermo Fisher Scientific, Waltham, MA), 20 ng/ml mouse stem cell factor (R&D Systems, Minneapolis, MN) and 40 pM basic fibroblast growth factor (Novus Biologicals, Littleton, CO). B16F1 cells were purchased from American Type Culture Collection (ATCC #CRL-6323; Manassas, VA) and grown in complete Dulbecco’s Modified Eagle’s Medium (DMEM, Invitrogen, Thermo Fisher Scientific). Expression vectors were transfected with X-tremeGENE HP (Sigma-Aldrich, St. Louis, MO) according to the manufacturer’s instructions.

Collagen gel solutions (2 mg/ml) were prepared by mixing rat type I collagen (Corning, Bedford, MA) with 10× minimal essential medium (MEM, final concentration of ×1, Gibco, Thermo Fisher Scientific), L-glutamine (2 mM final concentration; Gibco), sodium bicarbonate (3.7 g/L final concentration; Gibco), and FBS (10% final concentration). To generate thick fibrillar collagen substrates, 200 μl of collagen solution (pH adjusted to 7.4 with 1 M NaOH) was applied to 35-mm glass bottom culture dishes (MatTek Corp., Ashland, MA) and allowed to polymerize for 1 hr at room temperature. Melb-a cells suspended in culture medium were added to the substrates and cultured overnight for next day live-cell morphological studies. For 3D migration assays, cells were resuspended in complete medium and mixed with the prepared collagen mixture; the pH was adjusted to 7.4 with 1 M NaOH. The collagen and cell mixture was spread onto 35 mm MatTek dishes and allowed to polymerize for 1 hr at 37 °C for 30 min. Complete medium was added to the gels, and the cells were assayed for motility the next day.

Phase contrast live images of Melb-a cells cultured on thick collagen substrates and B16F1 cells cultured in the 3D collagen matrices were captured with the Eclipse TS100 Inverted Microscope with a 20×/0.40 Ph1 ADL objective lens (Nikon).

### Expression plasmids, viral constructs, and infection

mCherry-Lifeact was purchased from Addgene (#54491; Cambridge, MA), and pEGFP-Myo10 was previously described^5^. To knockdown mouse *MYO10* expression, we used the pLKO.1-TRC lentiviral vector (MISSION shRNA, Sigma-Aldrich) containing shRNA against mouse *MYO10* (shRNA-1;TRCN0000110606, shRNA-2;TRCN0000110608, and shRNA-3;TRCN0000379126). A non-target shRNA vector (SHC016; Sigma-Aldrich) was used as a control for the shRNA experiments. For lentiviral packaging, psPAX2 and pMD2.G vectors (Addgene plasmids #12260, #12259) were co-transfected with the pLKO.1 into HEK293T cells with X-tremeGENE HP (Sigma-Aldrich) according to the manufacturer’s instructions. Virus-containing medium was harvested 72 hrs after transfection and filtered with 0.45 μm filters (EMD Millipore, Billerica, MA). Melb-a or B16F1 cells were infected with culture supernatants from HEK293T cells at a 1:6 dilution in complete culture medium. Cells were passed after 24 hrs and selected with 4 μg/ml puromycin for 14 days.

### Immunoblotting

For kidney and brain, small portions excised from one-month-old mice were weighed and added to pre-heated Laemmli sample buffer (50 mM Tris, pH 7.4, 2% SDS, 6% glycerol, 0.1 M DTT and 0.25% bromophenol blue) at 500 μL/20 mg wet tissue weight and boiled for 15 min. Samples were homogenized with a Potter-Elehjem tissue grinder and passed through a 25 G needle with syringe 10 times. For induced dorsal skin melanomas, tumors were excised from euthanized mice and quickly frozen in liquid nitrogen. Samples were ground with a mortar and pestle in the presence of liquid nitrogen to create a powder. Pre-heated Laemmli sample buffer at 500 μL/20 mg wet tissue weight was added to the powdered tissue, and the sample was boiled for 15 min then homogenized with a 25 G needle and syringe. For cultured cells, cells grown in 6-well dishes were washed with PBS, collected with 100 μL Laemmli sample buffer, boiled for 15 min, and homogenized with a 25 G needle and syringe. All the lysates were centrifuged at 14,000 × g for 30 min at room temperature, and the supernatant was applied to SDS gels for separation followed by immunoblotting. To adjust the loading volume, serial dilutions of each lysate were subjected to SDS-PAGE using 4–12% Bis-Tris gels and then the gels were stained with Coomassie Brilliant Blue (CBB). After gel electrophoresis, lysates were transferred to nitrocellulose membranes at 4 °C in 20 mM Tris, 200 mM glycine, and 20% methanol using a Mini Trans-Blot cell (Bio-Rad) set to 100 V for 70 min, followed by blocking the nitrocellulose for 30 min in blocking buffer (PBS containing 0.1% Tween 20 with 5% dried milk). Membranes were then incubated overnight with 1 μg/mL anti-Myo10 antibody (Sigma #HPA024223) in blocking buffer. After washing three times for 5 min in washing buffer (PBS containing 0.1% Tween 20) membranes were incubated with goat anti-rabbit IgG (H + L)-HRP conjugate (Bio-Rad) diluted (1:15,000) in blocking buffer for 30 min. Following five 5-min washes, membranes were incubated with Amersham ECL Prime Western Blotting Detection Reagent (GE Healthcare) and imaged on Amersham Hyperfilm (GE Healthcare). For the loading control, HRP was inactivated by incubating with 30% (w/v) hydrogen peroxide at 37 °C for 15 min. Then, after re-blocking the membranes were re-probed with mouse monoclonal anti-α-tubulin antibodies (Sigma, T5168) and developed with ECL reagent as above. In the case of the tissue lysates, probed membranes were stained with CBB to control for the amount loaded and transfer efficiency. Images on films and CBB-stained membranes were captured with a photo scanner (Canon, Pixma) and converted to gray scale by Photoshop Elements 13 (Adobe Systems).

### Immunocytochemistry and 3D live-cell imaging

B16F1 cells cultured in 3D collagen matrices were fixed with 4% PFA for 15 min and permeabilized in PBS containing 0.1% Triton X for 10 min. After blocking with blocking buffer (5% BSA in PBS) for 30 min, anti-Myo10 antibody or anti-VASP rabbit monoclonal antibody (9A2, Cell Signaling Technology) diluted in blocking buffer was applied to 3D cultures overnight at 4 °C. After washing with PBS, cells were incubated with Alexa Fluor 488 goat anti-rabbit IgG (Thermo Fisher Scientific) and Alexa Fluor 594 phalloidin (Invitrogen) in blocking buffer for 1 h. Cells were then washed three times with PBS and mounted in Vectashield mounting medium with DAPI (Vector Laboratories, Burlingame, CA). Fluorescence images were captured on the TCS SP5 confocal microscope with an HCX PL APO 40×/1.25 objective lens (Leica) using sequential acquisition to give separate image files for each. A scan speed of 400 Hz and 4-frame averaging were used, and gain and offset were adjusted to give sub-saturating fluorescence intensity with optimal signal-to-noise ratio.

3D live-cell imaging was performed using a Zeiss LSM 710 confocal microscope with an EC Plan-Neofluor 40×/1.30 objective lens (Carl Zeiss, Jena, Germany). Image capture and processing were performed using the ZEN 2012 (Zeiss) software package. B16F1 cells co-transfected with GFP-Myo10 and mCherry-Lifeact were cultured in 3D collagen matrices 16 hrs before the experiments and maintained at 5% CO_2_ and 37 °C in a temperature-controlled chamber. Time-lapse images were obtained with a 1,024 × 1,024 (xy) pixel scan field (pixel size: 0.16 μm) by sequential epifluorescent illumination with 1.58 μsec pixel dwell and 5-min intervals over 30 min. Captured images were exported to ImageJ and reconstructed to Z-stack images from an 8 μm Z-range with 17 slices (0.5 μm intervals) using maximum intensity projection.

### Transwell migration assay

A total of 5 × 10^4^ Melb-a melanoblasts suspended in 0.5% FBS with complete medium was seeded into the upper chamber inserts (8.0 μm, 24 well) of Transwells (Corning, Bedford, MA). The top chamber inserts were placed in the bottom wells, which were filled with 5% FBS with complete medium, overnight. The non-migrating cells present on the upper surface of the insert were removed with a cotton swab. The cells that migrated onto the lower surface of the filter were fixed and stained with 0.1% crystal violet. The number of migrated cells in 4 different random fields on each membrane was counted using ImageJ software. Each experiment was repeated at least three times.

### Experimental metastasis

Experimental metastasis was performed as described previously with slight modification^74^. Briefly, B16F1 cell lines were trypsinized and suspended in PBS. Cells (2 × 10^5^) were then injected intravenously into the tail veins of 5–7 week old female C57BL6 mice. Two weeks after injection, the mice were euthanized, the lungs were removed, and the black spherical colonies were counted. In total, 10 mice were used for control shRNA cells, and 10 mice were used for Myo10 shRNA cells.

### *In silico* data analysis

cDNA microarray data sets of malignant melanoma and normal tissue samples were analyzed using the Oncomine database (www.oncomine.org). The effect of Myo10 expression in malignant melanoma on patients’ overall survival was analyzed by using the online survival analysis tool PROGgeneV2 (http://watson.compbio.iupui.edu/chirayu/proggene/database/). The cohort was divided into two groups based on median expression of Myo10 to analyze the correlation between Myo10 expression and overall survival. The Kaplan-Meier plots, logrank p value, hazard ratio, lower and upper confidence intervals of hazard ratio displayed in the survival plot legend were also generated using PROGgeneV2. Using the cBioPortal database (www.cbioportal.org), the cohort from TCGA-SKCM was divided two groups with (*n* = 55) or without (*n* = 405) *TP53* mutation in melanoma patients’ samples. The data of *MYO10* mRNA expression were downloaded and analyzed.

### Statistical analysis

All statistical analyses (except Oncomine and PROGgeneV2 data analyses) were performed with Prism 6 (GraphPad Software, San Diego, CA). Unpaired t-test was used for comparing data between two groups. *P* < 0.05 was considered significant.

## Electronic supplementary material

Supplementary Information

Supplementary Movie S1

Supplementary Movie S2
