# Supplementary material for: Myosin X is required for efficient melanoblast migration and melanoma initiation and metastasis
Source: Sci Rep. 2018 Jul 11;8:10449. doi: 10.1038/s41598-018-28717-y (PMC6041326; doi:10.1038/s41598-018-28717-y)

Myosin X is required for efficient melanoblast migration and melanoma initiation and metastasis

Hiroshi Tokuo[1]*, Jag Bhawan[2], and Lynne M. Coluccio[1]

[1]Department of Physiology & Biophysics, Boston University School of Medicine, Boston, MA 02118 USA; and

[2]Department of Dermatology, Boston University School of Medicine, Boston, MA 02118 USA

*Corresponding author

Address correspondence to:

Hiroshi Tokuo, M.D., Ph.D.
Department of Physiology & Biophysics
Boston University School of Medicine
700 Albany Street
Boston, MA 02118-2518
Tel: 617-358-8765
Fax: 617-638-4041
E-mail: tokuo@bu.edu

Supplementary Figure S1

The presence and zygosity of the *Myo10*$^{tm2(KOMP)Wtsi}$ allele were verified by PCR using genomic DNA isolated from tail snips. An image of the whole gel is shown.

Supplementary Figure S2

Immunoblotting of cell lysates from Melb-a cell lines expressing control or Myo10-specific shRNA. An image of the whole immunoblot for Myo10 is shown in the upper panel and for tubulin is shown in the lower panel.

Supplementary Figure S3

Immunoblotting of cell lysates from B16F1 cell lines expressing control or Myo10-specific shRNA. An image of the whole immunoblot for Myo10 is shown in the upper panel and for tubulin is shown in the lower panel.

Supplementary Movie S1

Time-lapse images (shown in Fig. 7B, upper panel) were converted into a movie. The images were taken at 5 min/frame and shown at 1 sec/frame (300 times the original speed).

Supplementary Movie S2

Images of GFP-Myo10 and mCherry-Lifeact in Supplementary Movie 1 were converted to green and red, respectively and combined for merged images.

Supplementary Figure S1 (for Figure 1-C)

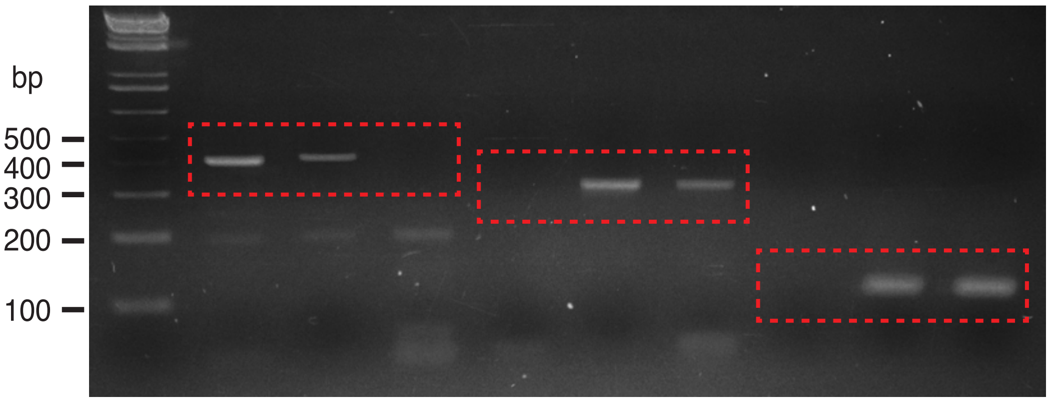

Figure S2 (for Figure 3-A)

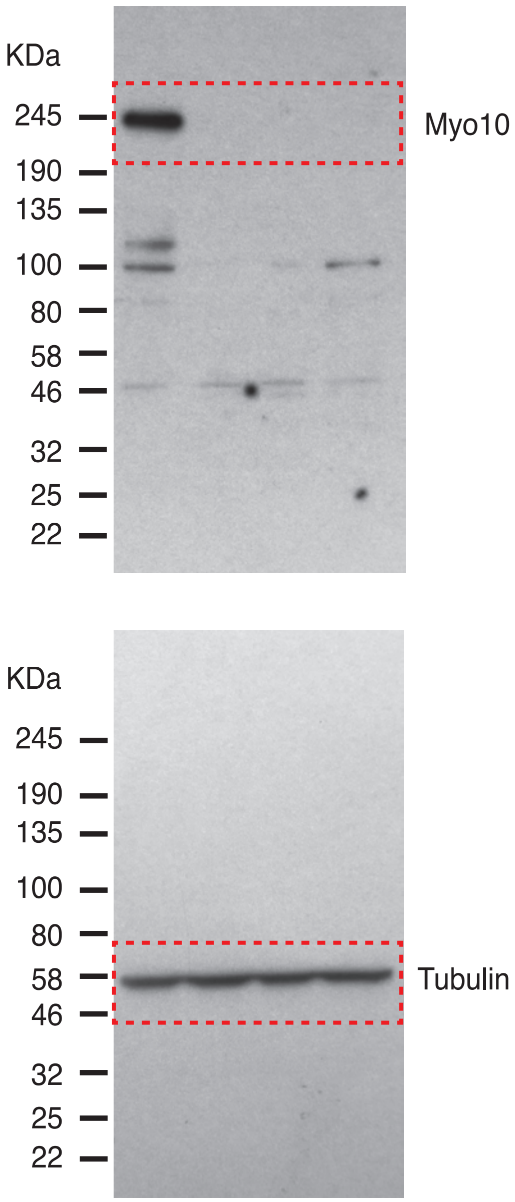

Figure S3 (for Figure 6-A)

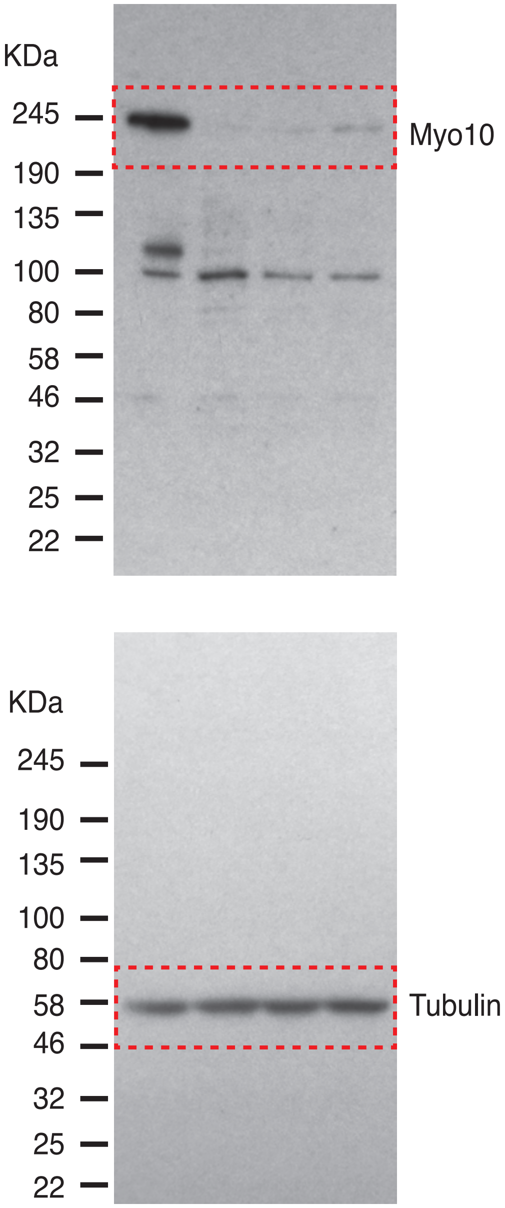

Supplement: Supplementary file 1 — Supplementary Information [file 41598_2018_28717_MOESM1_ESM.pdf]
